# Species Discrimination of *Stomoxys* Flies *S. bengalensis*, *S. calcitrans*, and *S. sitiens* (Diptera: Muscidae) Using Wing Geometric Morphometrics

**DOI:** 10.3390/ani13040647

**Published:** 2023-02-13

**Authors:** Tanasak Changbunjong, Tanawat Chaiphongpachara, Thekhawet Weluwanarak

**Affiliations:** 1 Department of Pre-Clinic and Applied Animal Science, Faculty of Veterinary Science, Mahidol University, Nakhon Pathom 73170, Thailand; 2 The Monitoring and Surveillance Center for Zoonotic Diseases in Wildlife and Exotic Animals (MoZWE), Faculty of Veterinary Science, Mahidol University, Nakhon Pathom 73170, Thailand; 3 Department of Public Health and Health Promotion, College of Allied Health Sciences, Suan Sunandha Rajabhat University, Samut Songkhram 75000, Thailand

**Keywords:** landmarks, morphometry, *Stomoxys bengalensis*, *Stomoxys calcitrans*, *Stomoxys sitiens*, vector

## Abstract

**Simple Summary:**

*Stomoxys* flies (Diptera: Muscidae) are bloodsucking insects that feed on both animals and humans. They are a major vector of a variety of animal pathogens, including agents of trypanosomosis, anaplasmosis, bovine leucosis, African horse sickness, lumpy skin disease, etc. This study investigated the effectiveness of landmark-based geometric morphometrics of wings to discriminate among three morphologically similar species of *Stomoxys* in Thailand: *Stomoxys bengalensis*, *Stomoxys calcitrans*, and *Stomoxys sitiens*. Our study reveals that geometric morphometrics can accurately discriminate the three species of *Stomoxys* based on wing shape. Therefore, wing geometric morphometrics can be used in addition to traditional morphological methods to identify these flies as species.

**Abstract:**

The flies of the genus *Stomoxys* Geoffroy, 1762 (Diptera: Muscidae), are regarded as pests of veterinary and medical importance. In Thailand, *Stomoxys calcitrans* (Linnaeus, 1758) is the most abundant species and is widely distributed throughout the country. This *Stomoxys* species can coexist with two other morphologically similar species: *Stomoxys bengalensis* Picard, 1908, and *Stomoxys sitiens* Rondani, 1873. Hence, discriminating using morphological characteristics is difficult, especially if the specimen is damaged or loses its diagnostic characteristics. In this study, we evaluated the effectiveness of the landmark-based geometric morphometric (GM) approach to discriminate among the three *Stomoxys* spp.: *S. bengalensis*, *S. calcitrans*, and *S. sitiens*. Left-wing images of *S. bengalensis* (*n* = 120), *S. calcitrans* (*n* = 150), and *S. sitiens* (*n* = 155) were used for the GM analyses. The results of the wing shape analyses revealed that the GM approach was highly effective for discriminating three *Stomoxys*, with high accuracy scores ranging from 93.75% to 100%. This study adds to the evidence that landmark-based GM is an excellent alternative approach for discriminating *Stomoxys* species.

## 1. Introduction

The genus *Stomoxys* Geoffroy, 1762, of the family Muscidae, subfamily Stomoxyinae, contains at least 18 described species, most of which are found in Africa, with some species also found in the Indian subcontinent and Southeast Asia [1]. *Stomoxys* species are all hematophagous ectoparasites of domesticated and wild animals, and occasionally humans. They have a direct impact on animal health and can even result in economic losses due to their effects on cattle productivity [2,3]. *Stomoxys calcitrans* (Linnaeus, 1758), the most well-known species with a global distribution, is an important vector of a variety of animal pathogens such as viruses, protozoa, bacteria, and helminths [2]. In Thailand, six *Stomoxys* species, namely, *Stomoxys bengalensis* Picard, 1908; *S. calcitrans*; *Stomoxys indicus* Picard, 1908; *Stomoxys pullus* Austen, 1909; *Stomoxys sitiens* Rondani, 1873; and *Stomoxys uruma* Shinonaga et Kano, 1966, as well as their distribution, have been recorded [4,5,6,7,8]. Among them, *S. calcitrans* was reported as the most predominant species [4,7], which was also involved in disease transmission, especially trypanosomosis or surra in horses, cattle, and buffaloes [9] and anaplasmosis in cattle and buffaloes [10]. Moreover, this species may be associated with epidemic outbreaks of lumpy skin disease in cattle throughout the country [11].

**Figure 1:**
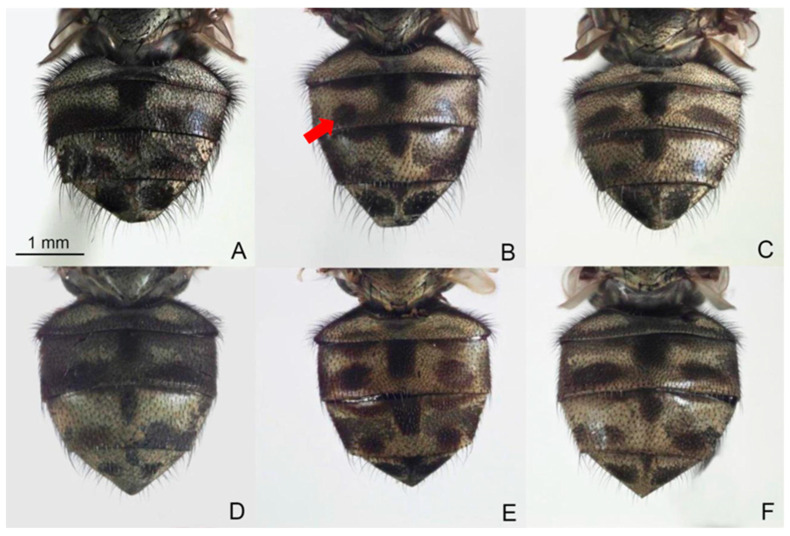
Male and female abdomens of *Stomoxys bengalensis* (**A**,**D**), *S. calcitrans* (**B**,**E**), and *S. sitiens* (**C**,**F**). The spot pattern (arrow) is used to distinguish among species. Photos are made by authors.

**Figure 2:**
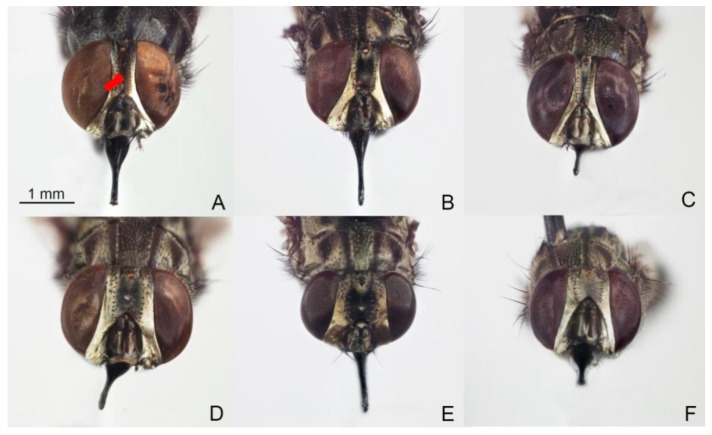
Male and female heads of *Stomoxys bengalensis* (**A**,**D**), *S. calcitrans* (**B**,**E**), and *S. sitiens* (**C**,**F**). The width of frons (arrow) is used to distinguish among species. Photos are made by authors.

**Figure 3:**
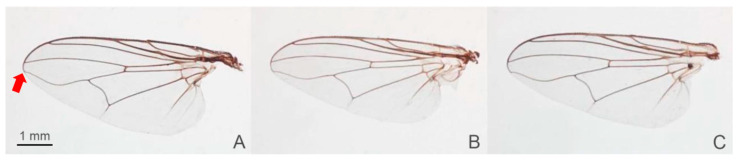
Wings of *Stomoxys bengalensis* (**A**), *S. calcitrans* (**B**), and *S. sitiens* (**C**). *Stomoxys bengalensis* is distinguished from *S. calcitrans* and *S. sitiens* by wing with the apex of media almost directly under the apex of r_4+5_ (arrow). Photos are made by authors.

**Figure 4:**
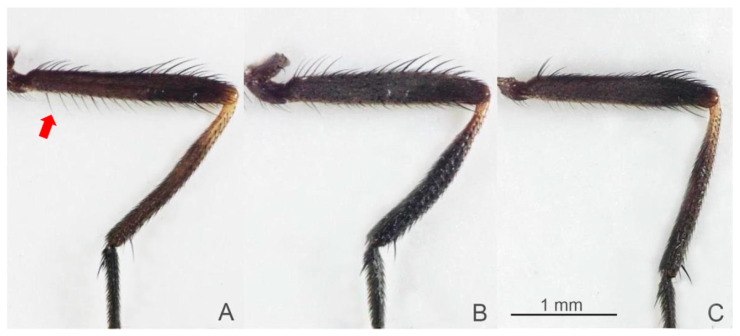
Male hind legs of *Stomoxys bengalensis* (**A**), *S. calcitrans* (**B**), and *S. sitiens* (**C**). Male *S. bengalensis* is distinguished from male *S. sitiens* by the hind femur with long ventral hairs (arrow). Photos are made by authors.

The precise species identification of *Stomoxys* is crucial for defining target vectors in the endemic area, which leads to the development of efficient vector control measures. However, the species identification of *Stomoxys* requires a thorough understanding of taxonomic characteristics. Traditionally, identification has been based primarily on morphological characteristics such as frons width, body color and pattern, wing cell proportion, curvature and setation of wing veins, color and hairs of legs, and genital structure [1]. Furthermore, the specimens must be clear and contain all relevant morphological features. Among the six *Stomoxys* spp. presented in Thailand, *S. calcitrans* is more similar to *S. sitiens* and *S. bengalensis* than to *S. indicus*, *S. pullus*, and *S. uruma* based on the spot pattern on the abdomen (Figure 1). According to Zumpt [1], *S. calcitrans* is distinguished from *S. sitiens* and *S. bengalensis* by the width of the frons (Figure 2), whereas *S. sitiens* and *S. bengalensis* are distinguished by the wing, with the apex of the media being slightly proximate to the apex of r_4+5_ in *S. sitiens*, which is almost directly under the apex of r_4+5_ in *S. bengalensis* (Figure 3). Moreover, the hind femur of male *S. bengalensis* has long ventral hairs that are not present in male *S. sitiens* (Figure 4). Therefore, if the important morphological features are damaged, it might be challenging to precisely identify them. To resolve problems associated with morphological-based identification of *Stomoxys* spp., alternative methods, such as the molecular method and geometric morphometrics, have gained popularity in the last decade [12,13,14].

Geometric morphometrics (GM) is a quantitative approach to insect species identification based on size and shape analyses [15,16,17]. It is a simple, rapid, and low-cost method compared with morphological and molecular identification methods, although it does require some laboratory environment and/or tools, and some taxonomic knowledge is needed to choose the best landmarks. In addition to being used for species identification, the GM method can be used to investigate intraspecific variation among insect populations and to determine sexual dimorphism [15]. In insects, the wing is usually used for GM analysis because wing veins provide well-defined landmarks or outlines appropriate for morphometrics [15,16]. Previously, Changbunjong et al. demonstrated the effectiveness of GM for the species identification of *Stomoxys* [14]. They revealed that both landmark and outline-based GM of the wing can discriminate among the three *Stomoxys* spp., namely, *S. indicus*, *S. pullus*, and *S. uruma* [14]. For other insects, GM has proved to be beneficial for species identification and/or discrimination of several important vectors, including blow flies [18], flesh flies [19], mosquitoes [20,21,22,23,24,25,26], sand flies [27,28], tsetse flies [29], horse flies [30], and triatomine bugs [31].

In this study, the landmark-based GM approach was used to evaluate its effectiveness in discriminating among the three *Stomoxys* spp., namely, *S. bengalensis*, *S. calcitrans*, and *S. sitiens*. The results of this study provide an alternative approach for identifying the species of these flies. Furthermore, our study material can be used as species reference data for morphometric identification of these species based on wing geometry.

## 2. Materials and Methods

### 2.1. Ethical Statement

This research protocol was checked, reviewed, and approved by the Faculty of Veterinary Science, Mahidol University Animal Care and Use Committee (Ethics Approval Number: MUVS-2022-01-04).

### 2.2. Specimen Collection and Species Identification

**Figure 5:**
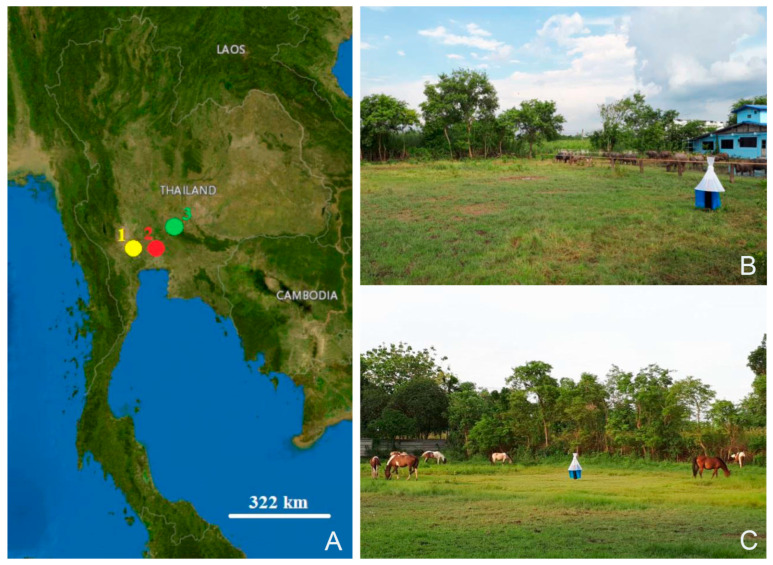
Geographic map of *Stomoxys* collection sites in this study: Nakhon Pathom (1), Pathum Thani (2), and Saraburi (3) (**A**); Vavoua trap used for fly collection and animal hosts at the collection sites (**B**,**C**). The map was created using the USGS National Map Viewer (public domain): http://viewer.nationalmap.gov/viewer/, accessed on 8 February 2023.

Between May 2022 and November 2022, specimens of *S. bengalensis*, *S. calcitrans*, and *S. sitiens* were collected from animal farms in three central Thai provinces, namely, Nakhon Pathom, Pathum Thani, and Saraburi, using five Vavoua traps [32] (Figure 5, Table 1). These traps were randomly placed near the animal hosts and enclosures and were used for four consecutive days during the day (06:00–18:00). All captured specimens were euthanized in a freezer at −10 °C, deposited in 1.5 mL microcentrifuge tubes separately, and delivered to the Vector-Borne Diseases Research Unit, Faculty of Veterinary Science, Mahidol University, Nakhon Pathom, Thailand.

Undamaged *Stomoxys* specimens with clearly morphological characteristics (“unambiguous specimens”) were used to identify species based on the descriptions and taxonomic keys of Zumpt [1] using a stereomicroscope (Nikon SMZ745; Nikon Corp., Tokyo, Japan). Zumpt [1] divided the key used to identify *Stomoxys* into males and females. Therefore, specimens for GM analysis were analyzed separately as male and female of each species. The specimens were then safely stored in a freezer at −20 °C until further GM analysis.

### 2.3. Specimen Preparation

The left undamaged wings of male and female *S. bengalensis*, *S. calcitrans*, and *S. sitiens* were dissected from the thorax using a sterilized blade and mounted on microscope slides using Hoyer’s medium [14]. Each wing slide was then photographed using a digital camera attached to a stereomicroscope (Nikon AZ 100; Nikon Corp., Tokyo, Japan). On each wing image, a 1-mm scale bar was placed. A total of 425 wing images (Table 1), including 155 wings of *S. sitiens*, 150 wings of *S. calcitrans*, and 120 wings of *S. bengalensis*, were analyzed using the landmark-based GM approach in the next step (Tables S1 and S2).

### 2.4. Wing Geometric Morphometric Analyses

#### 2.4.1. Landmark Digitization and Digitization Error

**Figure 6:**
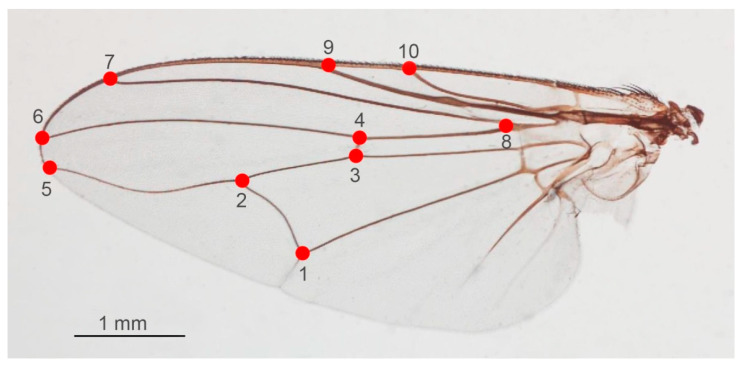
Ten anatomical landmarks (red dots and numbers from 1 to 10) digitized on the wing of *Stomoxys* flies for the landmark-based GM analysis.

According to the previous study by Changbunjong et al. [14], the coordinates of ten wing landmarks at the intersections of wing veins and wing boundaries were digitized (Figure 6). The repeatability index, which was computed using the Procrustes analysis of variance (ANOVA) method, was used to evaluate the accuracy and error for repeatability of the landmark digitization [33]. To assess intra- and inter-user repeatability, ten wing images of each *Stomoxys* species were chosen at random and digitized twice by the same and different users. In this study, if the repeatability index of wing shape was less than 90%, all wing images were re-digitized to ensure the precision of the coordinates [30].

#### 2.4.2. Wing Size Analyses

To estimate the wing size of male and female *S. bengalensis*, *S. calcitrans*, and *S. sitiens*, the centroid size (CS) was calculated by taking the square root of the sum of the squared distances between the centroid and each landmark [34]. Boxplots were constructed to illustrate the wing CS variation for each *Stomoxys* species. Moreover, the statistically significant difference in wing size between *Stomoxys* species was determined using a one-way ANOVA. The statistical significance was calculated using a non-parametric test (1000 permutations) with a Bonferroni correction at *p*-value of 0.05.

#### 2.4.3. Wing Shape Analyses

After performing a Procrustes superimposition using the generalized Procrustes analysis [35], the wing shape variables were subsequently obtained. Their principal components (PCs) were used as the final shape variables in wing-shape studies. The superposition of the mean wing shape provided configurations for comparing shape changes among species and sexes visually. Following that, the final wing shape variables were used as data for multivariate discriminant analysis (DA) [15], as shown by the factor maps illustrating wing shape variation among *Stomoxys* species. After DA, Mahalanobis distances, distance shape metrics that measure distances between species, were calculated. The statistically significant difference in wing shape based on Mahalanobis distances was determined using a non-parametric permutation test (1000 permutations) with a Bonferroni correction at *p*-value of 0.05.

#### 2.4.4. Validated Classification Based on Size and Shape

To determine the percentage of specimens correctly classified within their respective species, a cross-validated classification was performed. Each individual specimen was gradually removed from the total number of specimens and allocated to the most probable group based on the maximum likelihood approach for wing size investigation [36] and the closest group based on Mahalanobis distance for wing shape investigation [37].

#### 2.4.5. Allometric Effect Analysis

The allometric effect, also known as the effect of size on shape variation, was estimated using linear regression between the CS (size variable) and the first PC of shape (shape variable), and then the determination coefficient (r^2^) was used to assess the influence.

#### 2.4.6. Morphometric Software

The online software XYOM (XY Online Morphometrics) version 2 was used to digitize landmarks, investigate statistical size and shape, and generate graphics output [17]. This software is freely available at https://xom.io/, accessed on 1 January 2023.

## 3. Results

### 3.1. Intra-and Inter-User Repeatability

The two image sets of measurements performed by the same user and images showed a high degree of intra-user repeatability for shape (repeatability index score = 98.5%, measurement error = 1.5%), while the same images performed by different users demonstrated high inter-user repeatability for shape (repeatability index score = 97.5%, measurement error = 2.5%).

### 3.2. Wing Size Variation

**Figure 7:**
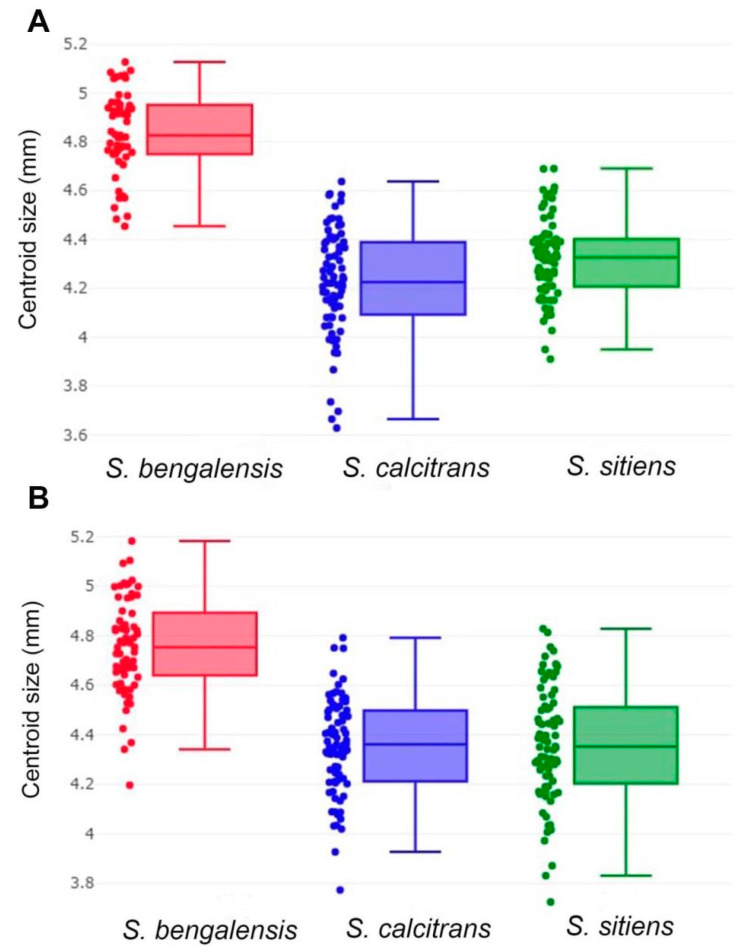
Boxplots of the centroid size variations of male (**A**) and female (**B**) *Stomoxys bengalensis*, *S. calcitrans*, and *S. sitiens*. The horizontal line that crosses each box represents the median, separating the 25th and 75th quartiles.

The wing size (CS) variation of male and female *S. bengalensis*, *S. calcitrans*, and *S. sitiens* is illustrated by quantile boxes (Figure 7). In males, *S. bengalensis* (4.83 ± 0.17 mm) had the largest wing, followed by *S. sitiens* (4.32 ± 0.16 mm) and *S. calcitrans* (4.23 ± 0.22 mm). Conversely, in females, *S. bengalensis* (4.76 ± 0.19 mm) had the largest wing, followed by *S. sitiens* (4.36 ± 0.23 mm) and *S. calcitrans* (4.35 ± 0.20 mm). Table 2 shows the statistically significant differences in wing CS among the three *Stomoxys* spp. in both sexes (*p* < 0.05).

### 3.3. Wing Shape Variation

**Figure 8:**
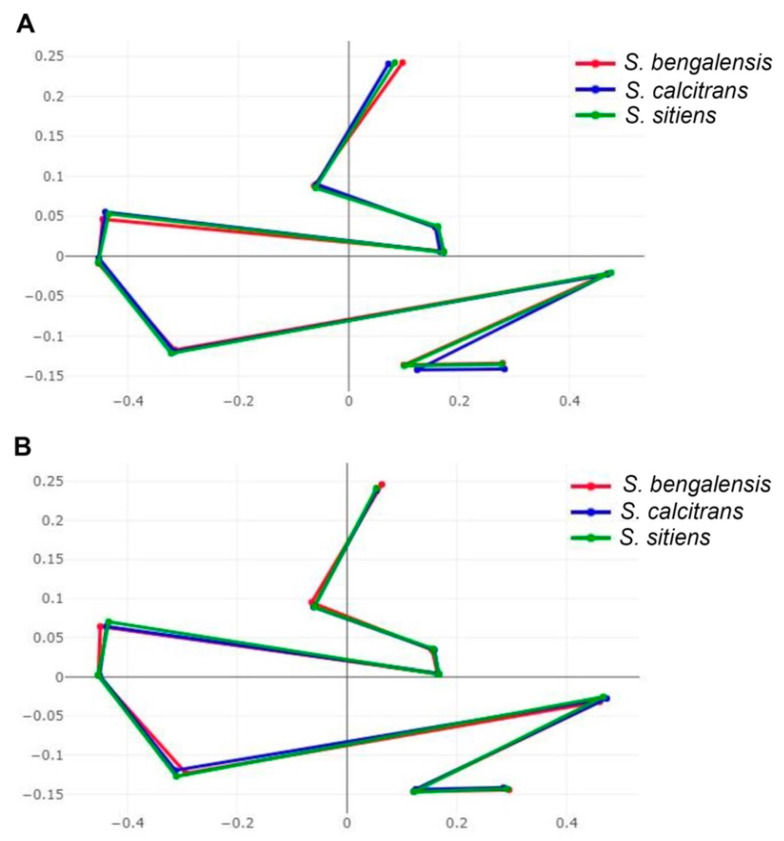
Superposition of the mean anatomical landmark positions of male (**A**) and female (**B**) *Stomoxys bengalensis*, *S. calcitrans*, and *S. sitiens*.

The visual comparisons of superposed configurations among the three *Stomoxys* spp. revealed landmark locations that differ greatly among species and are found in the upper, middle, and lower parts of the wing (landmarks 1, 5, 9, and 10) for males but only in the middle part of the wing for females (landmarks 5 and 7) (Figure 8).

**Figure 9:**
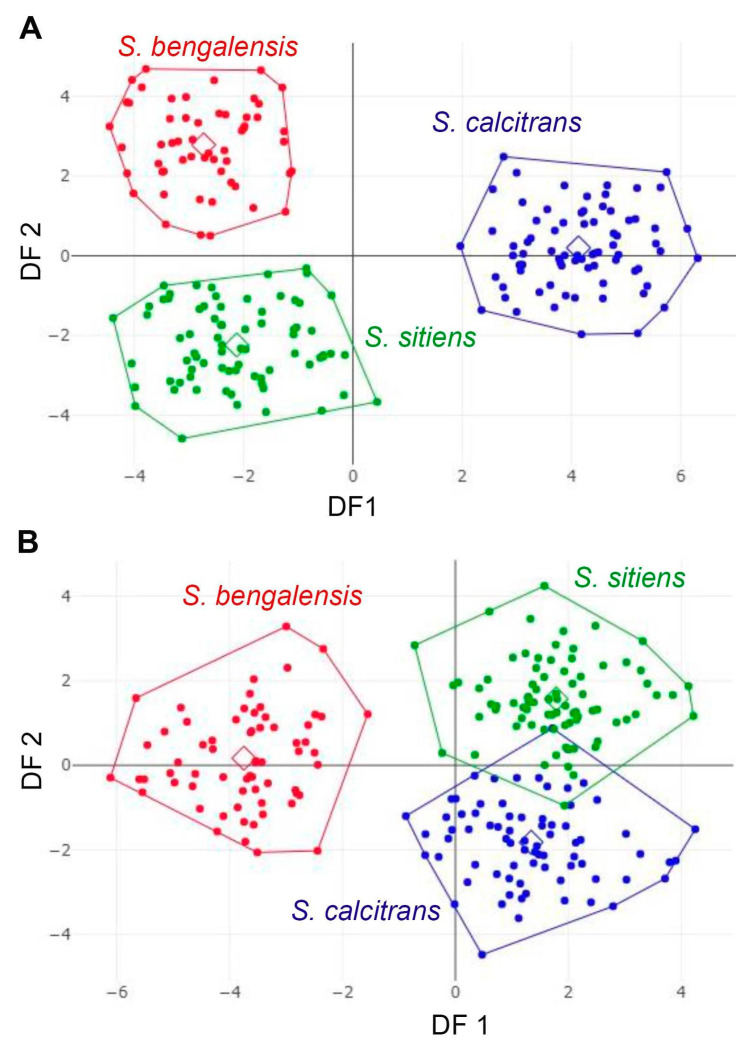
Factor maps of discriminant factors (DFs) showing wing shape variation in male (**A**) and female (**B**) *Stomoxys bengalensis*, *S. calcitrans*, and *S. sitiens*. For males, the two DFs represent 100% of the total discriminant space: 72% for DF1 and 28% for DF2. However, for females, the two DFs represent 100% of the total discriminant space: 74% for DF1 and 26% for DF2.

The factor maps of the first two shape-derived discriminant factors (DFs) revealed that the male groups of *S. bengalensis*, *S. calcitrans*, and *S. sitiens* are clearly separated from each other. In contrast, female groups of *S. calcitrans* and *S. sitiens* exhibited considerable overlapping (Figure 9). Further, the pairwise Mahalanobis distances of wing shape were significantly different among the three *Stomoxys* spp. in both sexes (*p* < 0.05, Table 3).

### 3.4. Validated Classification

The overall accuracy scores of size- and shape-based cross-validated classification of male and female *S. bengalensis*, *S. calcitrans*, and *S. sitiens* were 58.35% (248/425) and 98.35% (418/425), respectively (Table 4). The accuracy scores of wing size-based classification using the maximum likelihood technique were relatively low in males (62.93%, ranging from 48.00% to 89.09%) and in females (54.10%, ranging from 13.33% to 86.15%, Table 4). However, the accuracy score of shape-based classification based on Mahalanobis distance was perfect in males (100%) and very high in females (96.82%, ranging from 93.75% to 100%).

### 3.5. Allometric Effect

**Figure 10:**
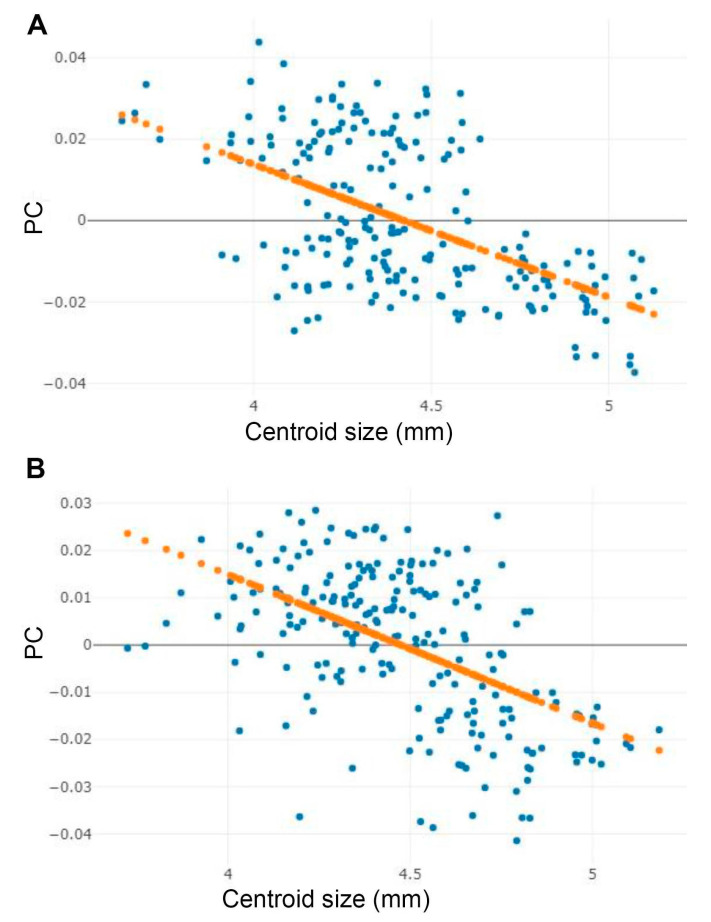
Linear regression between centroid size and first principal component (PC) of shape for the male (**A**) and female (**B**) *Stomoxys* species. Orange dotted line indicates linear regression prediction.

The analysis of the effect of wing size on shape variation in male and female among the three *Stomoxys* spp. showed that wing size was significantly correlated with wing shape variation (r^2^ = 30.6% for male and r^2^ = 29.2% for female, *p* < 0.05). The linear regression prediction of the allometric effect revealed negative correlations in both sexes (Figure 10).

## 4. Discussion

In this study, we demonstrate that the landmark-based GM approach is efficient for discriminating among the three *Stomoxys* spp. in Thailand, namely, *S. bengalensis*, *S. calcitrans*, and *S. sitiens*. These *Stomoxys* flies are morphologically similar and can be difficult to discriminate based on morphology alone. Therefore, modern methods for identifying the correct species of *S. bengalensis*, *S. calcitrans*, and *S. sitiens* are required. Among those species, *S. calcitrans* is the most abundant species, which can coexist with *S. bengalensis* and *S. sitiens* [8,38]. Therefore, it can be seen that most specimens used in this study were collected from the same site. In terms of species distribution, *S. calcitrans* and *S. sitiens* have been recorded in various geographical regions of Thailand [4,7,38], while *S. bengalensis* has been recorded predominantly in the central part of the country, such as Bangkok, Nakhon Pathom, Phra Nakhon Si Ayutthaya, and Saraburi Provinces [6,8,38]. The presence of these three *Stomoxys* species in the same area makes species confirmation even more difficult due to the inability to make predictive decisions.

The application of GM to the discrimination of *Stomoxys* spp. was initiated by Changbunjong et al. [14]. Their results revealed that both landmark- and outline-based methods can discriminate among the three *Stomoxys* spp., namely, *S. pullus*, *S. uruma*, and *S. indicus* [13]. In the present study, we selected ten anatomical landmark positions according to the previous study [14]. These positions have been proven to be useful in discriminating among the three *Stomoxys* spp. and can be used to investigate the phenotypic variation [39]. Additionally, 15 landmarks were used to investigate the wing size and shape variations of *S. calcitrans* under the influence of larval densities and substrate types during the larvae stage [40].

The comparison of wing size variation using mean CS showed that the wing size of *S. bengalensis* was significantly larger than that of *S. calcitrans* and *S. sitiens* in both sexes. Our findings indicated that the wing size could help in discriminating *S. bengalensis* from *S. calcitrans* and *S. sitiens.* However, due to its relatively low classification accuracy in terms of validated classification scores, we do not recommend using wing size for discrimination. Furthermore, wing size is regarded as unsuitable for taxonomic purposes because it is often influenced by environmental factors [39,40]. For instance, Baleba et al. [40] revealed that larval density and substrate quality had a significant effect on the wing size variation of *S. calcitrans*. The previous study by Chaiphongpachara et al. [39] also indicated that the phenotypic variation of *S. calcitrans* populations in Thailand has evolved in response to local environmental pressures.

The DA of wing shape variables showed that all males are clearly separated into each species, whereas only females of *S. calcitrans* and *S. sitiens* showed partial overlap in the factor map. These findings could be related to males having more easily identifiable morphological characteristics than females. Zumpt [1] mentioned that male *S. calcitrans* is easy to distinguish from other species in the genus through the broad frons, while male *S. bengalensis* can be easily distinguished from male *S. sitiens* through the presence of long ventral hairs on the hind femur. In contrast, the width of the frons of female *S. calcitrans* is not much different from the other two species, making identification difficult. Furthermore, the abdominal and wing venation patterns of *S. sitiens* are quite similar to those of *S. calcitrans* (Figure 1 and Figure 3). Therefore, they are occasionally misidentified. The results of the validated classification based on wing shape supported the misidentification of *S. calcitrans* and *S. sitiens*, with two specimens (2.67%) of *S. calcitrans* classified as *S. sitiens* and five specimens (6.25%) of *S. sitiens* classified as *S. calcitrans*. It is not surprising that the geometry of the wing shape could recognize *S. bengalensis* perfectly (100%), as evidenced by its separation from other species in the factor map (Figure 9).

The assessment of the allometric effect revealed a correlation between wing size and wing shape among the three *Stomoxys* spp. The linear regression showed a negative correlation, which is consistent with the findings of Chaiphongpachara et al. [39]. They found a negative correlation between wing size and wing shape in both sexes of *S. calcitrans* populations in Thailand (r^2^ = 24.0% for males and r^2^ = 15.0% for females). The linear regression displayed a negative correlation (also known as an inverse correlation), which means that one variable tends to increase while the other tends to decrease, or vice versa. However, the allometric effect was not removed from our study because size variations were considered important for the species identification process [15,24,41].

Overall, the landmark-based GM approach is nearly 99% accurate in discriminating the three *Stomoxys* spp. (*S. bengalensis*, *S. calcitrans*, and *S. sitiens*) when compared with the same method used to discriminate other *Stomoxys* spp. (*S. pullus*, *S. uruma,* and *S. indicus*), with an accuracy of approximately 93% [14]. We also found that digitizing *Stomoxys* wings provided excellent precision, regardless of whether the user was the same (98.5% intra-user repeatability) or different (97.5% inter-user repeatability). Therefore, our study material (landmark coordinate data) can serve as reference data for morphometric identification of these species. Finally, we suggest that a landmark-based GM approach can be used as a complement to traditional morphology identification, especially for *Stomoxys* specimens with unclear diagnostic characteristics or those that have been damaged during specimen collection and handling in the field. Moreover, this method is inexpensive and fast and does not require an expert taxonomist for these flies.

## 5. Conclusions

In this study, we evaluated the effectiveness of the landmark-based GM approach to discriminate among the three *Stomoxys* spp., namely, *S. bengalensis*, *S. calcitrans*, and *S. sitiens*. Our results demonstrated that this method was highly effective in discriminating them based on their wing shape. As a result, when specimens have ambiguity or loss of diagnostic characteristics, the GM can be used as a supplement to morphological identification. In addition, our findings suggest that our study material can be used as reference data to assist in species identification. Therefore, we included the wing geometric morphometric data of *S. bengalensis*, *S. calcitrans*, and *S. sitiens* in the supplementary material for interested researchers to download and use. The use of the GM approach is important for accurate identification of *Stomoxys* species and may lead to more effective control measures for the flies in animal farms.

## Figures and Tables

**Table 1:** Collection sites, month, and number (*n*) of *Stomoxys* specimens used for the geometric morphometric analysis.

Species	Month	District/Province	Region	Collection Sites	*n*
*S. bengalensis*	May	Mueang, Nakhon Pathom (N 14°01′10″, E 99°57′37″)	Central	Cattle farm and horse farm	Male 45, Female 45
	July	Sam Khok, Pathum Thani (N 14°04′46″, E 100°31′51″)	Central	Cattle farm	Male 10, Female 20
*S. calcitrans*	May	Mueang, Nakhon Pathom (N 14°01′10″, E 99°57′37″)	Central	Cattle farm and horse farm	Male 25, Female 25
	July	Sam Khok, Pathum Thani (N 14°04′46″, E 100°31′51″)	Central	Cattle farm	Male 25, Female 25
	November	Kaeng Khoi, Saraburi (N 14°28′39″, E 101°05′33″)	Central	Horse farm	Male 25, Female 25
*S. sitiens*	May	Mueang, Nakhon Pathom (N 14°01′10″, E 99°57′37″)	Central	Cattle farm and horse farm	Male 50, Female 50
	November	Kaeng Khoi, Saraburi (N 14°28′39″, E 101°05′33″)	Central	Horse farm	Male 25, Female 30
Total					425

**Table 2:** Mean centroid size of male and female *Stomoxys bengalensis*, *S. calcitrans*, and *S. sitiens* and statistically significant differences.

Species	*n*	Mean (mm)	Min–Max	Variance	SD
Male					
*S. bengalensis*	55	4.83 ^a^	4.45–5.13	0.03	0.17
*S. calcitrans*	75	4.23 ^b^	3.63–4.64	0.04	0.22
*S. sitiens*	75	4.32 ^b^	3.91–4.69	0.03	0.16
Female					
*S. bengalensis*	65	4.76 ^a^	4.20–5.18	0.04	0.19
*S. calcitrans*	75	4.35 ^b^	3.77–4.79	0.04	0.20
*S. sitiens*	80	4.36 ^b^	3.72–4.83	0.05	0.23

Different letters after the mean value represent statistically significant differences in wing CS between male and female (*p* < 0.05).

**Table 3:** Mahalanobis distances (below diagonal) and *p*-values (above diagonal) in the wing shape of male and female *Stomoxys bengalensis*, *S. calcitrans*, and *S. sitiens*.

Species	*S. bengalensis*	*S. calcitrans*	*S. sitiens*
Male			
*S. bengalensis*	-	<0.001	<0.001
*S. calcitrans*	7.33	-	<0.001
*S. sitiens*	5.06	6.71	-
Female			
*S. bengalensis*	-	<0.001	<0.001
*S. calcitrans*	5.46	-	<0.001
*S. sitiens*	5.71	3.42	-

**Table 4:** Cross-validated classification based on the wing sizes and wing shapes of male and female *Stomoxys bengalensis*, *S. calcitrans*, and *S. sitiens*.

Species	Size		Shape	
	% Accuracy	Assigned/Observed	% Accuracy	Assigned/Observed
Male				
*S. bengalensis*	89.09	49/55	100	55/55
*S. calcitrans*	58.67	44/75	100	75/75
*S. sitiens*	48.00	36/75	100	75/75
Total	62.93	129/205	100	205/205
Female				
*S. bengalensis*	86.15	56/65	100	65/65
*S. calcitrans*	13.33	10/75	97.33	73/75
*S. sitiens*	66.25	53/80	93.75	75/80
Total	54.10	119/220	96.82	213/220

## Data Availability

The data presented in this study are available in Supplementary Materials.

## Supplementary Materials

The following supporting information can be downloaded at: https://www.mdpi.com/article/10.3390/ani13040647/s1, Table S1: Raw coordinates of male wing landmarks of *Stomoxys bengalensis* (No. 1–55), *S. calcitrans* (No. 56–130), and *S. sitiens* (No. 131–205). Table S2: Raw coordinates of female wing landmarks of *Stomoxys bengalensis* (No. 1–65), *S. calcitrans* (No. 66–140), and *S. sitiens* (No. 141–220).
